# Localization of *Staphylococcus aureus* in tissue from the nasal vestibule in healthy carriers

**DOI:** 10.1186/s12866-017-0997-3

**Published:** 2017-04-05

**Authors:** Anne-Merethe Hanssen, Bert Kindlund, Niels Christian Stenklev, Anne-Sofie Furberg, Silje Fismen, Renate Slind Olsen, Mona Johannessen, Johanna Ulrica Ericson Sollid

**Affiliations:** 1 Research Group for Host-Microbe Interaction, Department of Medical Biology, Faculty of Health Sciences, UiT - The Arctic University of Norway, N-9037 Tromsø, Norway; 2 Sahlgrenska Academy, University of Gothenburg, Gothenburg, Sweden; 3 Department of Clinical Medicine, Faculty of Health Sciences, UiT - The Arctic University of Norway, Tromsø, Norway; 4 Department of Microbiology and Infection Control, University Hospital of North Norway, Tromsø, Norway; 5 Department of Community Medicine, Faculty of Health Sciences, UiT - The Arctic University of Norway, Tromsø, Norway; 6 Department of Pathology, University Hospital of North-Norway, Tromsø, Norway; 7 Division of Drug Research, Department of Medical and Health Sciences, Faculty of Health Sciences, University of Linköping, Linköping, Sweden; 8 Department of Laboratory Medicine, Division of Medical Diagnostics, Jönköping, Region Jönköping County Sweden

**Keywords:** Nasal colonization, *Staphylococcus aureus*, Carrier, Localization, Epithelial cells, Intracellular

## Abstract

**Background:**

Colonization of the body is an important step in *Staphylococcus aureus* infection. *S. aureus* colonizes skin and mucous membranes in humans and several animal species. One important ecological niche of *S. aureus* is the anterior nares. More than 60% of the *S. aureus* in the nose are found in vestibulum nasi. Our aim was to describe the localization of *S. aureus* in nasal tissue from healthy carriers.

**Methods:**

Punch skin biopsies were taken from vestibulum nasi from healthy volunteers (*S. aureus* carriers and non−/intermittent carriers, *n* = 39) attending the population-based Tromsø 6 study. The tissue samples were processed as frozen sections before immunostaining with a specific *S. aureus* antibody, and finally evaluated by a confocal laser-scanning microscope.

**Results:**

Our results suggest that *S. aureus* colonize both the upper and lower layers of the epidermis within the nasal epithelium of healthy individuals. The number of *S. aureus* in epidermis was surprisingly low. Intracellular localization of *S. aureus* in nasal tissue from healthy individuals was also detected.

**Conclusions:**

Knowledge of the exact localization of *S. aureus* in nasal tissue is important for the understanding of the host responses against *S. aureus.* Our results may have consequences for the eradication strategy of *S. aureus* in carriers, and further work can provide us with tools for targeted prevention of *S. aureus* colonisation and infection.

**Electronic supplementary material:**

The online version of this article (doi:10.1186/s12866-017-0997-3) contains supplementary material, which is available to authorized users.

## Background

The nose is an important site for *Staphylococcus aureus* and methicillin-resistant *S. aureus* (MRSA) colonization in humans, and *S. aureus* is predominantly located in the anterior nasal vestibule on the septum adjacent to the nasal ostium on the moist squamous epithelium [1, 2]. In this location, *S. aureus* can exist as a commensal without any signs or symptoms of an infection. The prevalence of persistent nasal carriage with *S. aureus* is 20–30% in adult human populations [3, 4], and most often it is an endogenous *S. aureus* strain that causes infection [5–7]. Prevention and elimination of the carrier state may contribute in reducing the *S. aureus* disease burden [4]. Successful nasal decolonization of *S. aureus* is difficult to achieve, and *S. aureus* readily recolonizes the nose, the throat and other sites within one week, commonly with the same *S. aureus* genotype [8–11]. Today, nasal mupirocin is the most efficacious regimen of *S. aureus* eradication from the anterior nares [12, 13]. However, the success rate for eradication of MRSA is low 12 months after decolonization [14].

The anterior part of the nasal cavity (vestibulum nasi) is lined with stratified, keratinized, squamous epithelium. More than 60% of *S. aureus* in the nasal vestibule are found here [1]. It has also been shown that *S. aureus* can bind to the ciliated nasal epithelial cells in the inner part of the nasal cavity (internal nares) with pseudostratified columnar ciliated epithelium [15–18]. The skin in vestibulum nasi consists of two main layers: epidermis and dermis [19]. Epidermis consists of five strata called the stratum corneum, stratum lucidum, stratum granulosum, stratum spinosum, and stratum basale [20, 21]. The five main strata are characterized by cells at varying stages of development. Stratum corneum, also called the cornified layer, is the outermost layer and contains mature keratinocytes called corneocytes [21, 22]. The stratum lucidum is a thin, translucent layer with no nuclei. Stratum granulosum are flattened keratinocytes which contain cytoplasmic granula and secretory organelles [21, 23]. Stratum spinosum consists of several layers of large polygonal keratinocytes, also called spinous keratinocytes. Stratum basale is the layer that separates dermis from epidermis, and it consists of undifferentiated epidermal cells. The cells in this layer are responsible for constant renewal of cells in epidermis [21]. Dermis is a layer of connective tissue and contains accessory epidermal structures, lymphatic and vascular conduits, nerves and nerve endings, collagen and elastic fibers, in addition to many specialized immune cells [20, 21].

Successful adhesion of microbes to the human host depends on an efficient combination of microbial surface components as well as human host ligands. *S. aureus* adhesion to human epithelium is mediated by wall teichoic acid (WTA) and microbial surface components recognizing adhesive matrix molecules (MSCRAMMs) [24, 25]. WTA is important for both initial and late phases of *S. aureus* colonization, while surface proteins are important for long term *S. aureus* persistence in the nasal cavity [25]. Clumping factor B (ClfB) [26, 27] and the iron-regulated surface determinant A (IsdA) [26, 28] are important for nasal colonization and adhere to cytokeratin and loricrin/involucrin in corneocytes [26, 27, 29, 30]. *S. aureus* adhesion is also dependent on the host phenotype [25, 31, 32], but very few ligands in the host are known. Recently, it was shown that the interaction between the serine-aspartate repeat protein D (SdrD) and the human desmosome protein, desmoglein 1, is important for adhesion to keratinocytes in vitro [33].

*S. aureus* not only adhere to cell surfaces during colonization, but also invade both phagocytic and non-phagocytic cells where they can survive antibiotic treatment [16, 34, 35]. Here, they constitute a significant risk factor for recurrent episodes of disease, e.g. rhinosinusitis [15, 16], tonsillitis [36] and osteomyelitis [37].

For developing new strategies to eradicate *S. aureus* and diminish the risk of invasive disease, it is crucial to understand the host-pathogen interaction in colonization, invasion and infection. Fundamental questions of these aspects include; where in the epithelial layer is *S. aureus* located and how does *S. aureus* interact with cells in the nasal vestibule in healthy individuals? Thus, our aim was to determine where *S. aureus* localize in the nasal epithelium, and to evaluate a possible intracellular residency.

## Methods

### Study design and population

In 2010 an invitation letter for participation in the study was sent to a random selection of men and women age 40–42 years (*n* = 193) with known *S. aureus* nasal carrier status in the sixth Tromsø study (Tromsø 6) from October 2007–August 2008, a large population-based multipurpose health study [38, 39]. The *S. aureus* carrier status of the participants included in this study was defined in Tromsø 6 [38, 39]. Our working definition for nasal carriage types was according to van Belkum et al. [4]. There are two types of human nasal *S. aureus* carriers: persistent carriers and others, i.e. noncarriers and intermittent carriers are grouped together. A total of 48 individuals (25% of the invited) were willing to participate in this cross-sectional sub-study in 2010–2011. Among these volunteers, we performed a selection based on *S. aureus* carriage results from Tromsø 6 with the purpose of recruiting approximately 50% carriers of *S. aureus* and 50% non−/intermittent carriers, with an equal distribution of women and men. The selection represents a homogenous population according to age, with small age-related tissue-changes. The *S. aureus* noncarriers were considered as normal controls. Before tissue sampling, the volunteers were interviewed by a physician concerning known allergies, medicinal use, and ongoing cold/upper respiratory infection or fever. Exclusion criteria included known allergies against local anesthetics, and current use of anticoagulants (warfarin, acetylsalicylic acid) or other medication that preclude tissue sampling. If the subject was undergoing antibiotic treatment, symptomatic respiratory infection or fever at the time of sampling, the donor was asked to return for sampling after resolution of symptoms. Thirty-nine (*n* = 39) volunteers were finally included for nasal biopsy sampling at the University Hospital of North Norway (UNN HF), i.e. 18 females and 21 males, 18 *S. aureus* carriers and 21 non- /intermittent carriers.

### Ethics statement

This work was performed in compliance with the ethical guidelines established by UiT- The Arctic University of Norway. Each participant gave written informed consent prior to the tissue sampling. Subject recruitment, enrollment and sampling of nasal tissue samples have been approved by the Regional Committee for Medical and Health Research Ethics, Rec North, Norway (Document reference 2010/146–7).

### Microbiological sampling for detection and *spa*-typing of *S. aureus*

After interviewing the participants, a microbiological swab sample was taken from one nostril for confirmation of *S. aureus* carriage according to procedures described earlier [39]. *S. aureus* isolates and MRSA were identified using standard bacteriological criteria and laboratory methods, and all *S. aureus* isolates were *spa*-typed according to Sangvik et al. [39].

### Tissue collection

A tissue sample was harvested from one nostril, while a microbiological sample was harvested from the other nostril. Selection of nostril (left or right side) for 3 mm punch biopsy sampling was done randomly by flipping a coin. Before tissue sampling, local anesthetic was injected (Xylocaine 1% infiltration anesthetic, 0.4 mm diameter hypodermic needle, approx. 1 ml) subcutaneously at the sampling site. Entry of injection cannula was at least 5 mm from sampling site. The thickness of the histological specimen was 2–4 mm. It included the whole of epidermis and parts of dermis. The biopsy was harvested from the transition zone between skin and mucosa in the upper lateral vestibulum nasi. In total, 33 samples were obtained from keratinized squamous epithelium without nasal hair and six samples from the same region containing nasal hair. The biopsy sampling was divided between two physicians.

### Embedding of tissue and sectioning of frozen sections

Immediately after tissue sampling the tissue was embedded in Frozen Section Medium (OCT) (Fisher Scientific/Thermo Scientific), frozen in precooled liquid isopentane, and stored at −80 °C. The frozen tissue was sectioned in 10 μm sections with a glass/cryostat knife. The biopsy was sectioned vertically from the dermal site towards the epidermis with a cryostat blade that was cleaned with alcohol after obtaining each section. We started sectioning from one side of the biopsy towards the middle of the biopsy where most of our sections were obtained. Sections were placed on Super Frost Plus glass slides (Thermo Scientific) for immunohistochemistry and ordinary glasses for Hematoxylin-Eosin (HE)-staining. Sections and remaining tissue were stored at −80 °C for further use.

### Fluorescence labeling/staining

To detect *S. aureus*, 10 μm cryostat sections of nasal tissue were fixed (4% Formaldehyde), blocked (2% Goat serum, Sigma-Aldrich; 0.1% cold Fish Skin Gelatin; 1% bovine serum albumin, Sigma-Aldrich; 0.1% Triton X-100; 0.05% Tween-20; diluted in 1X phosphate buffered saline PBS, ThermoFisher Scientific), and incubated with rabbit polyclonal *S. aureus* antibody (ab20920, Abcam) 1:5000 dilution over night at 4 °C followed by Alexa Fluor 546® goat anti-rabbit IgG (A-11010, Molecular Probes™, Thermo Fisher Scientific) 1:1000 dilution labeling, or Alexa Fluor 488® goat anti-rabbit IgG (A-11008, Molecular Probes™, Thermo Fisher Scientific) 1:1000 dilution labeling for 30–60 min. The slides were incubated with DRAQ5 1:1000 (BioStatus) to stain keratinocyte nuclei, and Alexa Fluor 594 Phalloidin (A12381, Molecular Probes™, Thermo Fisher Scientific) 1:40 dilution for staining actin red. The samples were dried and the glass slides mounted by adding Prolong Gold Antifade reagent with DAPI (P36935, Molecular Probes™, Thermo Fisher Scientific). Samples were stored at +4 °C before immediate microscopy.

### Tissue analysis/microscopic analysis

Slides were analyzed by confocal microscopy using a Zeiss confocal microscope LSM510 Meta (Carl Zeiss Microscopy GmbH, Germany), with ×63 or ×40 objectives. Images were captured using the LSM510-imaging software system, version 4.2.0.121 (Carl Zeiss Inc.). For determining intracellular localization we used the Zeiss confocal microscope LSM510 Meta (Carl Zeiss Microscopy GmbH, Germany) using the LSM510 imaging software or Leica TCS SP5 (Leica Microsystems CMS GmbH, Germany) using the LAS AF software, version 2.3.0. For inspection of intracellular residency, multiple consecutive images were taken in the axial z-axis of tissue samples with a slice thickness of 0.2 μm or 0.5 μm. The tissue sections were assessed for evidence of *S. aureus,* visualized as bright green coccoid structures fluorescing on stimulation with a 488-nm laser. Samples were categorized as intracellular positive if *S. aureus* could be identified in at least one z-plane and closely localized adjacent to intraepithelial nuclei.

The relative amount of *S. aureus* present in the nasal tissue was calculated by randomly selecting minimum 10 consecutive tissue sections from 14 donors (eight carriers and six non−/intermittent carriers randomly selected), labeling the sections with *S. aureus* specific antibody, performing confocal laser scanning microscopy (CLSM) and calculating the mean number of *S. aureus* per tissue section. We counted all positive green cocci. Microscope images/slides were read and analyzed by two independent, blinded observers (RSO and AMH) for the presence of *S. aureus* in epidermis and intracellular localization.

### Hematoxylin-eosin (HE) staining

We fixed 10 μm tissue sections in 4% formaldehyde and immersed with hematoxylin (Harris, Thermo Scientific), stained in Scott’s solution (NaHCO_3_, MgSO_4_, and H_2_O), and immersed in eosin (Thermo Scientific). The sections were dehydrated in 96% ethanol and 100% ethanol, immersed in xylene (Sigma-Aldrich), and finally mounted onto glass slides with Histokit (Fisher Scientific) before examination in light microscope. The HE stained sections were used to investigate histopathological alterations.

### Gram-staining

For identification of Gram-positive cocci, frozen sections were stained following standard Gram-staining procedures (Sigma Aldrich). The Gram-stained sections were examined by light microscopy.

### Bacterial controls and antibody specificity

To avoid cross-reaction with *Staphylococcus epidermidis* and to test the specificity of the *S. aureus* polyclonal antibody we included bacterial controls in the immuno-fluorescence staining. We used *S. aureus* ATCC 25923 as positive control for primary antibody binding, and *S. epidermidis* ATCC 12228 as negative control. Overnight cultures of the controls were made in 5 ml Brain-Heart Infusion broth. 1 ml o.n. culture was centrifuged for 10 min at 3000 rpm, the supernatant was removed and the pellet resuspended in 1 ml 1× PBS. Centrifugation was repeated for 10 min at 3000 rpm, the pellet was resuspended in 1 ml 1× PBS, and 1 μl suspension was spotted on a glass slide, before flame fixation. From this point, the positive and negative controls were prepared in exactly the same way as the frozen tissue sections. Green fluorescence was detected from the positive controls only.

### Determination of *S. aureus* colony forming units (CFU) in the nose of healthy volunteers

To estimate the likelihood of detecting *S. aureus* in nasal tissue biopsies from healthy individuals, we performed a pilot study where 14 volunteers (colleagues working at the Department for Medical Biology, UiT – The Arctic University of Norway) were sampled by nasal swabbing according to Sangvik et al. 2011 [39], and the *S. aureus* CFU in the nose was determined. One nostril of each volunteer was swabbed in the anterior region, 1–1.5 cm inside the nostril. The swab was resuspended thoroughly in 1 ml of 0.9% sodium chloride. Twenty microliters from each tenfold dilution (10^0^–10^8^) was applied onto blood agar plates and ChromID *S. aureus* agar (Biomerieux) and incubated at 37 °C for 24 h. Colonies were counted in spots from two dilutions (3 if possible). The CFU/nostril was calculated, both total CFU and *S. aureus* CFU.

## Results

### *S. aureus* Carrier status versus results from confocal microscopic analyses and nasal swabbing

Thirty-nine healthy volunteers were included in the study, and their carrier status was determined in the previous Tromsø 6 study, i.e. 18 carriers and 21 non−/intermittent carriers. According to one swab sampling in this study, 16 out of 39 individuals were positive for *S. aureus* (Table 1), while 23 out of 39 individuals were negative for *S. aureus*. No MRSA were detected. *S. aureus* was observed in biopsy sections by CLSM in ten out of 18 persistent carriers (Table 1). Among the 21 non−/intermittent carriers, we found *S. aureus* in three of the biopsies by CLSM (Table 1). Thus, detection of *S. aureus* by one nasal swabbing and CLSM showed some inconsistency, i.e. three individuals that were negative for *S. aureus* by swabbing, both in the Tromsø 6 study and in the present study, were positive for *S. aureus* by CLSM, whereas three individuals with positive swab cultures in both studies, were negative for *S. aureus* by CLSM (Table 1). Two individuals defined as non−/intermittent carriers in the Tromsø 6 study, were positive for *S. aureus* by one swabbing, but negative by CLSM in our study (Table 1). Four individuals that were defined as persistent *S. aureus* carriers in the Tromsø 6 study were negative for *S. aureus* in our study based on swabbing and CLSM analysis (Table 1). Seven out of 39 biopsies were not examined in our study due to damaged sections, which was most likely caused by tissue sampling errors or sectioning errors (Table 1, not determined).

**Table 1:** Results from nasal swab culture and confocal laser scanning microscopy (CLSM) of tissue sections

*S. aureus* carriage status in the Tromsø 6 study^a^ (*n* = 39)	Presence of *S. aureus* by one nasal swab sampling present study^c^ (*n* = 39)		Presence of *S. aureus* by CLSM present study^d^ (*n* = 39)	
Persistent carriers (*n* = 18)	+	14	+	10
			−	3
			nd	1
	−	4	+	0
			−	4
			nd	0
Others^b^ (*n* = 21)	+	2	+	0
			−	2
			nd	0
	−	19	+	3
			−	10
			nd	6

^a^Results from nasal swabbing (two samplings with median time interval of 28 days) and *spa*-typing in the Tromsø 6 study in October 2007–August 2008. *S. aureus* carriage status as defined by van Belkum et al. [4]

^b^Includes both noncarriers and intermittent carriers

^c^Results from one nasal swab sampling and *spa*-typing in the present study in 2010–2011; +, positive for *S. aureus*; −, negative for *S. aureus*

^d^Confocal laser scanning microscopy (CLSM); +, positive for *S. aureus*; −, negative for *S. aureus*; nd, not determined, CLSM not performed

Fourteen different *spa*-types were found among the *S. aureus* isolates from the 39 healthy participants in this study (Additional file 1: Table S1). Twelve *spa*-types were found in one individual each, while t084 and t246 were found in two individuals each. Six individuals had changed *spa*-types since the Tromsø 6 study (Additional file 1: Table S1).

### Estimation of *S. aureus* in nasal vestibule in healthy individuals

We performed a pilot study to estimate the likelihood of detecting *S. aureus* in biopsies. From nasal swabbing of 14 healthy colleagues, we found between 0 and 1.5 × 10^4^ *S. aureus* CFU/nostril (Table 2). By a rough estimate, this should correspond to approximately 0–200 *S. aureus* per biopsy surface area.

**Table 2:** Number of *S. aureus* found in nares of healthy individuals

Number of *S. aureus* CFU/nostril (*n* = 14)^a^					
*n* = 8	*n* = 2	*n* = 1	*n* = 1	*n* = 1	*n* = 1
0	50	1.5 × 10^2^	5.0 × 10^2^	6.0 × 10^2^	1.5 × 10^4^

^a^Results from a pilot study where we determined *S. aureus* CFU/nostril in 14 healthy colleagues from Department of Medical Biology by one nasal swab culture

**Fig. 1:**
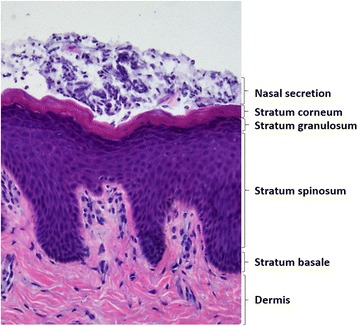
Representative histological view of epidermis in an *S. aureus* nasal carrier. Nasal secretion and four stratified cell layers in epidermis are marked. Sections were stained with hematoxylin-eosin, and examined in light microscope

Biopsies were then obtained from the 39 participants and HE staining and CLSM/immunohistochemistry (IHC) were performed. The cryo-sectioning of nasal tissue showed all layers of epidermis, confirming the quality of the biopsies (Fig. 1).

### Localization of *S. aureus* in epidermis and in nasal secretions

**Fig. 2:**
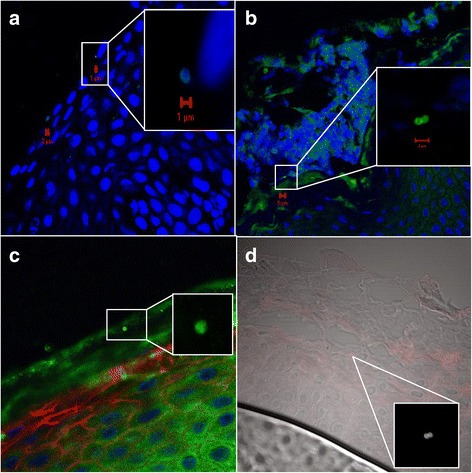
Localization of *S. aureus* in the upper part of epidermis. Inset represents a zoomed portion of the image. Scale bar is in micrometers. We used a confocal laser scanning microscopy (CLSM) ×63 objective. **a-b** *S. aureus* is localized on the border between stratum corneum and stratum granulosum. Green fluorescence labeling of *S. aureus*, blue fluorescence labeling of keratinocyte cell nuclei and *S. aureus* DNA. Primary antibody rabbit polyclonal antibody to *S. aureus* (ab20920, Abcam), secondary antibody Alexa Fluor 488® goat anti-rabbit IgG (Molecular Probe™, Thermo Fisher Scientific), and DRAQ5 (BioStatus). **c** *S. aureus* in stratum corneum. The same labeling is used as in a-b, but in addition, we used Alexa Fluor 594 Phalloidin (A12381, Molecular Probes™, Thermo Fisher Scientific) staining actin red. **d** Combined CLSM and light microscopy of nasal tissue section. *S. aureus* seen as red cocci in pairs, presumably on the border between stratum corneum and stratum granulosum. Primary *S. aureus* rabbit polyclonal antibody (ab20920, Abcam) and a secondary antibody Alexa Fluor 546 goat anti-rabbit IgG (Molecular Probes™, Thermo Fisher Scientific) were used

**Fig. 3:**
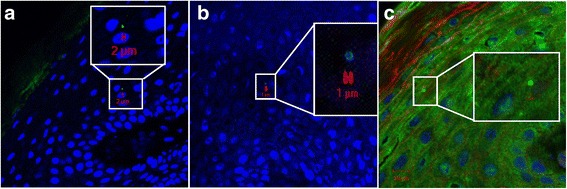
Localization of *S. aureus* in stratum spinosum by CLSM. Inset represents a zoomed portion of the image. Scale bar is in micrometers. We used a CLSM ×63 objective. The sections are oriented so that the outermost epidermis layer is shown in the upper left corner. **a-b** Green fluorescence labeling of *S. aureus*, blue fluorescence labeling of keratinocyte cell nuclei and *S. aureus* DNA. **c** Same labeling as in a and b, but in addition we used red fluorescence labeling of actin. Primary antibody rabbit polyclonal antibody to *S. aureus* (ab20920, Abcam), secondary antibody Alexa Fluor 488® goat anti-rabbit IgG (Molecular Probe™, Thermo Fisher Scientific), Alexa Fluor 594 Phalloidin (Molecular Probes™, Thermo Fisher Scientific) and DRAQ5 (BioStatus)

**Fig. 4:**
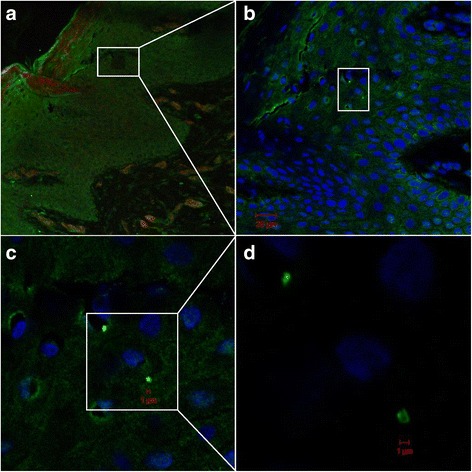
Confocal laser scanning microscopy of frozen section of nasal tissue from an *S. aureus* carrier. Overview (**a-b)** and detailed/zoomed portion of image (**c-d)** showing *S. aureus* in stratum spinosum. Scale bar is in micrometers. Green fluorescence labeling of *S. aureus*, blue fluorescence labeling of keratinocyte cell nuclei and *S. aureus* DNA, red fluorescence labeling of actin. Primary antibody rabbit polyclonal antibody to *S. aureus* (ab20920, Abcam), secondary antibody Alexa Fluor 488® goat anti-rabbit IgG (Molecular Probe™, Thermo Fisher Scientific), Alexa Fluor 594 Phalloidin (Molecular Probes™, Thermo Fisher Scientific) and DRAQ5 (BioStatus). We used a CLSM ×63 objective

**Fig. 5:**
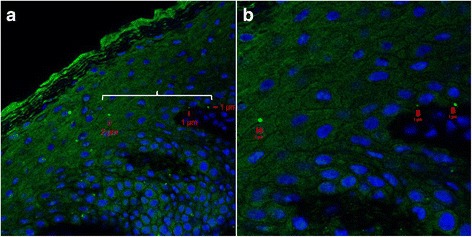
Localization of *S. aureus* on the border between stratum spinosum and stratum basale. Overview (**a)** and detailed image (**b)** of nasal tissue from an *S. aureus* carrier by confocal laser scanning microscopy. Scale bar is in micrometers. White brace indicates the region which is magnified in (b). Green fluorescence labeling of *S. aureus*, blue fluorescence labeling of keratinocyte cell nuclei and *S. aureus* DNA. Primary antibody rabbit polyclonal antibody to *S. aureus* (ab20920, Abcam), secondary antibody Alexa Fluor 488® goat anti-rabbit IgG (Molecular Probe™, Thermo Fisher Scientific), and DRAQ5 (BioStatus). We used a CLSM ×63 objective

**Fig. 6:**
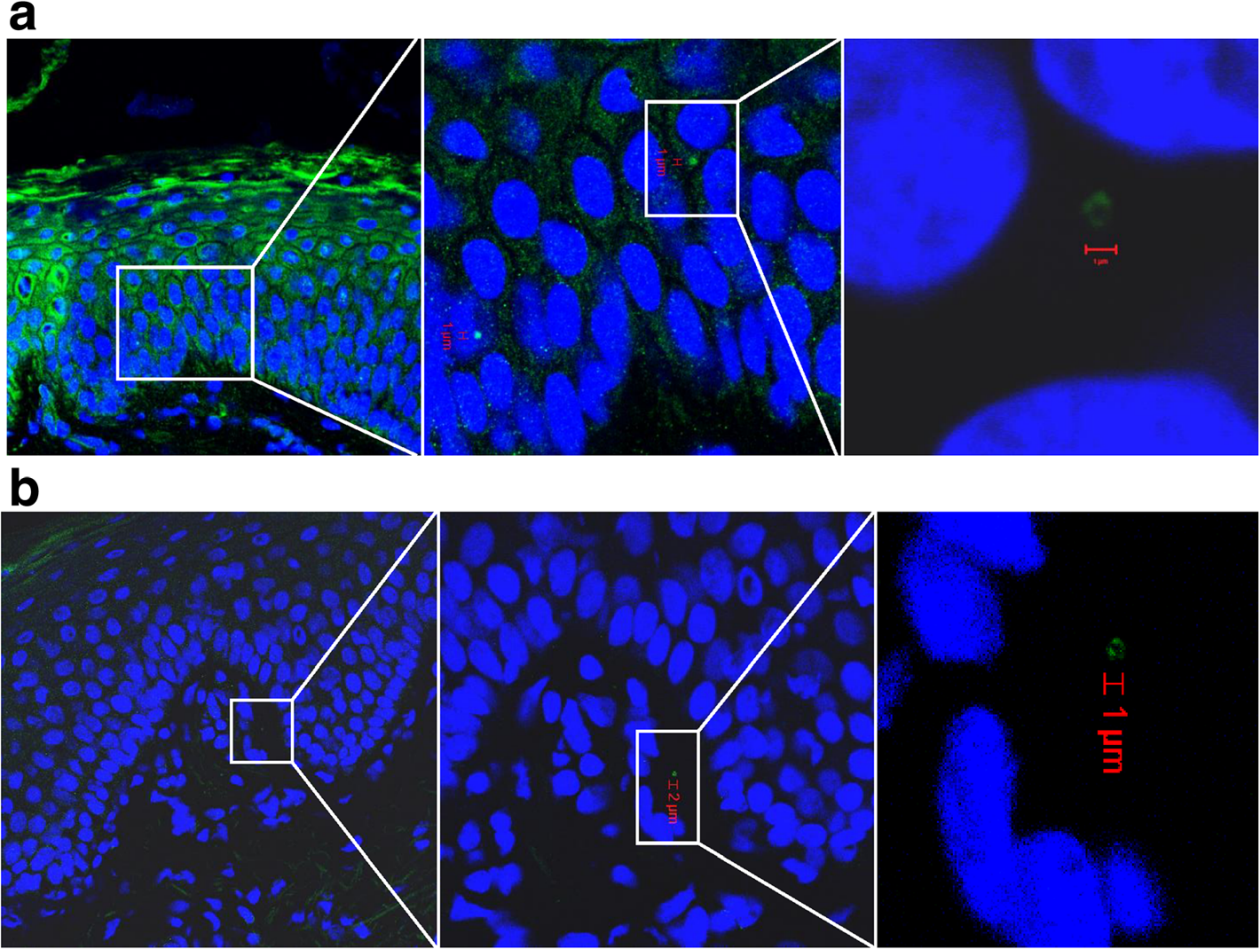
Localization of *S. aureus* in the lower part of epidermis by CLSM. Overview and detailed image/zoomed portion of the image showing (**a**) *S. aureus* on the border between stratum spinosum and stratum basale, and (**b**) *S. aureus* on the border between the stratum basale and dermis. Inset represents a zoomed portion of the image. Scale bar is in micrometers. Green fluorescence labeling of *S. aureus*, blue fluorescence labeling of keratinocyte cell nuclei and *S. aureus* DNA. Primary antibody rabbit polyclonal antibody to *S. aureus* (ab20920, Abcam), secondary antibody Alexa Fluor 488® goat anti-rabbit IgG (Molecular Probe™, Thermo Fisher Scientific), and DRAQ5 (BioStatus). We used a CLSM ×63 objective

We detected *S. aureus* in both the upper and lower layers of epidermis by CLSM/IHC (Figs. 2, 3, 4, 5 and 6). *S. aureus* was most often found in stratum corneum (Fig. 2). We also found *S. aureus* in stratum spinosum (Figs. 3, 4 and 5), as far down as in stratum basale (Figs. 5 and 6), and on the border to dermis (Fig. 6b). Nasal secretion was observed in sections from both carriers and non−/intermittent carriers, and *S. aureus* was often observed in association with nasal secretion in pairs or clusters (Fig. 2b). *S. aureus* was often observed as single bacterial cells (Fig. 2a and c, Figs. 3, 4, 5 and 6) or in pairs (Fig. 2b and d) in the epidermal layers. Gram-staining confirmed the presence of Gram-positive cocci in the epidermis of both carriers and non−/intermittent carriers (results not shown).

**Fig. 7:**
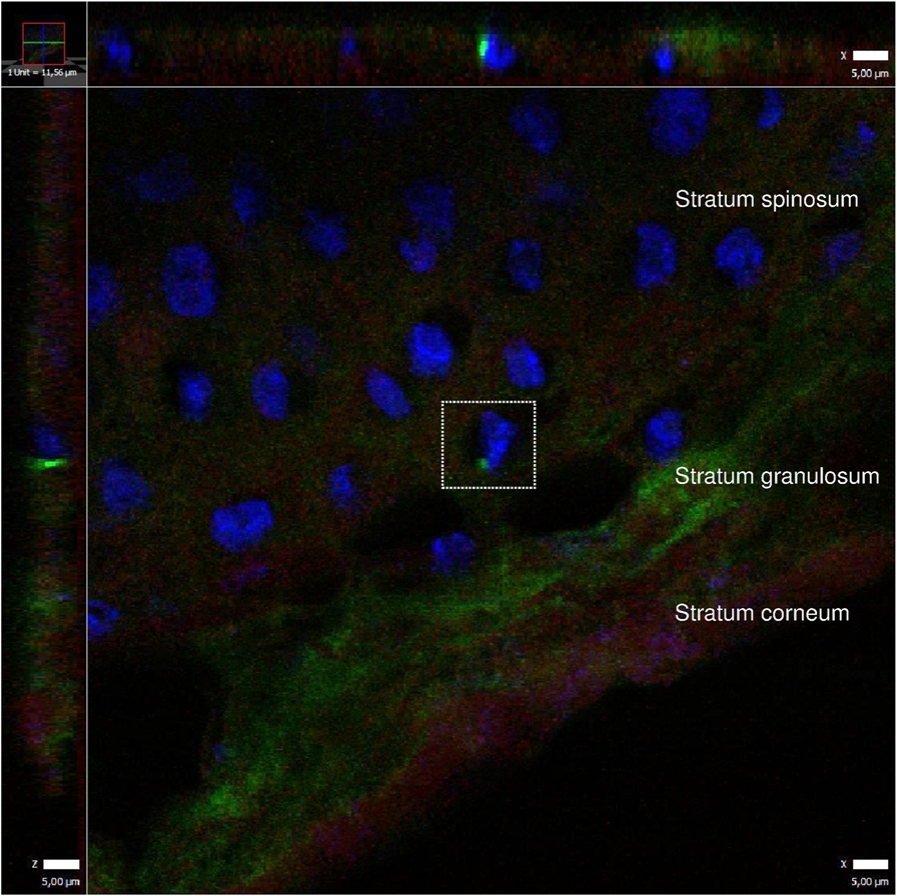
Intracellular localization of *S. aureus* in nasal epithelial cells. *S. aureus* is labeled with primary rabbit polyclonal antibody to *S. aureus* (Abcam), secondary antibody Alexa Fluor 488 goat anti-rabbit IgG (green) (Molecular Probes™, Thermo Fisher Scientific), DRAQ5 (BioStatus) for keratinocyte nuclei (blue) and Alexa Fluor 594 Phalloidin (A12381, Molecular Probes™; Thermo Fisher Scientific) for actin (red). Confocal laser scanning microscopy of frozen sections. Projection is constructed from confocal Z-stacks (0,2 um thick), 63× objective. Image to the left and on top corresponds to a vertical view in the z-plane. Z-plane images reveal a single cellular nucleus (blue) closely related to fluorescing *S. aureus* (green)

Intracellular localization of *S. aureus* was assessed by CLSM/IHC (Fig. 7) using specific antibodies to *S. aureus* and staining of different structures (nucleus and actin). Three-dimensional reconstruction of highly magnified z-stacks was possible using CLSM. Consecutive z-plane images (Z-scan) showed fluorescent *S. aureus* (green) in close proximity/closely localized to the cellular nuclei of epithelial cells (blue) (Fig. 7).

Among the 39 biopsy donors, we selected 14 donors randomly for calculating the number of *S. aureus* per tissue section by CLSM/IHC. The counting was performed by blinding the sample numbers and the examiner was unaware whether a carrier or non−/intermittent carrier was examined when performing the confocal microscopy. On average, there were 0.61 *S. aureus* per tissue section among all the 14 selected individuals in our study. Of note, *S. aureus* was detected in sections from three of the six selected non−/intermittent carriers, while no *S. aureus* was detected in the blinded sections from three out of eight *S. aureus* carriers.

## Discussion

In the present study, we have assessed the *S. aureus* localization within vestibulum nasi in healthy individuals. By using a confocal scanning laser microscopy approach, we found that *S. aureus* is localised in both the upper and lower layers of epidermis within the nasal epithelium of healthy individuals. We also observed intracellular localization of *S. aureus* in the different layers of epidermis.

As expected, we identified the majority of *S. aureus* in the stratum corneum. In artificially colonized cotton rats, localization of *S. aureus* has been shown in the stratum corneum of intranasal squamous epithelium [24] where *S. aureus* appeared as single cells or diplococci, a finding that is corroborated by our study. This is also in accordance with other studies showing that adherence of *S. aureus* to nasal cells is directly related with the age of the epithelial cells. The most differentiated cells bind most *S. aureus*, while the youngest cells (less differentiated keratinocytes in the lower epidermis layer) bind fewer *S. aureus* [40]. Identical findings have been shown for *S. epidermidis* [41].

A histological study revealed *S. aureus* in the stratified squamous epithelium and in hair follicles in vestibulum nasi post mortem [42]. The study suggested that there is a difference between persistent and intermittent *S. aureus* carriers concerning niche localization, and difficulties in decolonization might be related to *S. aureus* confined to hair follicles and subsequent re-colonization. We took samples from regions with nasal hair in only six donors, but unfortunately we did not observe hair follicles in any of these samples.

The number of *S. aureus* captured from swabbing of the nasal vestibule is quite low [1], but the number of *S. aureus* in the nostrils is in accordance with other studies [1, 26, 43]. As expected, the low density of *S. aureus* was confirmed by the microscopic analyses. Normally, there are higher bacterial densities in diseased tissue compared with tissue from healthy carriers [16]. A previous study using confocal microscopy on tissue sections from rhinosinusitis patients, found that approximately 1/3 of *S. aureus* infected cells (regardless of cell type) carried more than 10 bacteria [16]. We confirmed our IHC/fluorescence staining protocol by using a skin tissue section from a patient with confirmed *S. aureus* skin infection as positive control (results not shown). In this positive control, we observed large clusters of *S. aureus* in both upper and lower layers of epidermis. Our findings show a clear difference between *S. aureus* colonization in healthy and inflamed vestibular skin, as the density of *S. aureus* was low in the healthy volunteers in our study.

The squames (stratum corneum) is constantly being shed from the skin surface, and shedding contributes to the clearing of attached bacteria [44]. This might explain why we saw only a few *S. aureus* in the nasal tissue sections. In many of our tissue sections, the stratum corneum was detached from the epithelial layer, and was visible as loose appendages in the proximity of the epithelium.

One important deviation in our study when defining colonization status, is that when we screen for *S. aureus*, we normally swab both nostrils [45], but in this study we only swabbed one of the nostrils and harvested our histologic sample from the other nostril. The reason for this was that we wanted to keep an intact skin structure and not alter the skin surface before biopsy sampling. It has previously been shown that carriers are more likely to carry *S. aureus* in one nostril than in both nostrils [46], and this could perhaps explain the cases where we detected *S. aureus* by swab culture, but not by CLSM of biopsied nasal tissue from the same individual, or vice versa.

We observed a switch of *S. aureus* colonization status in the period between the Tromsø 6 study and our study in some of the participants. Three individuals defined as non−/intermittent carriers in the Tromsø 6 study, were negative for *S. aureus* by nasal swab sampling and positive for *S. aureus* by CLSM of nasal epithelium in the present study. We also observed the opposite, i.e. four individuals defined as *S. aureus* carriers in the Tromsø 6 study were *S. aureus* negative in our study by both swabbing and CLSM. A change in colonization status of some individuals was expected [39]. Another expected observation was that some persistent carriers exhibited different *spa* types in the two studies. These observations are in line with earlier reports [39, 47], and could be examples of strain replacement or co-colonization.

Intracellular persistence of *S. aureus* in connection with infection, e.g. recurrent sinusitis, has been shown in previous studies [15, 16]. We confirmed intracellular *S. aureus* localization in the stratum spinosum in healthy individuals by CLSM images at multiple z-planes within the nasal tissue by using specific antibodies. We did not perform a systematic investigation of intracellular localization and it was only performed in a few randomly selected donors. *S. aureus* can live as a commensal within humans without causing overt disease, and there is considerable evidence of intracellular localization of *S. aureus* [16, 34, 35] in neutrophils [48], osteoblasts [49], nasal epithelial cells [16, 50], and endothelial cells [51]. However, we did not expect to find intracellular *S. aureus* in nasal tissue from healthy individuals.

The intracellular localization of *S. aureus* has been suggested to provide a sanctuary for the bacteria, protecting them from the innate host defense system, and perhaps also from antimicrobial agents [16], hence serving as a reservoir for chronic or relapsing infection. Some of the participants in our study were negative by *S. aureus* nasal swabbing, but positive for *S. aureus* by CLSM indicating that intracellular localization of *S. aureus* could be difficult to detect by nasal swabbing. A possible explanation for the discrepancy could be intracellular localization or it could be due to a single nostril *S. aureus* carrier status.

Our work shows that *S. aureus* is primarily observed in the debris overlying the nasal epithelium, whereas single staphylococci can also be observed in deeper intraepithelial layers. We observed nasal secretions in most of the tissue samples from both carriers and non−/intermittent carriers. Clusters of *S. aureus* were very often localized within the nasal secretions in carriers, and especially in two of our donors. One could hypothesize that staphylococci are in a dormant state in their intraepithelial localization. In the healthy nasal vestibule, the stratum corneum is not surveilled by the dendritic cells [52], which could mean that staphylococci might remain undetected by the body’s immune system. In addition, nasal crust formation could be considered a natural growth medium for staphylococci, leading to colony formation under the right host conditions.

## Conclusions

Our results suggest that *S. aureus* colonize both the upper and lower layers of the epidermis within the nasal epithelium of healthy individuals. The number of *S. aureus* in epidermis was surprisingly low. Intracellular localization of *S. aureus* in nasal tissue from healthy individuals was also detected. The intracellular state may protect the bacteria from traditional decolonization procedures and cause problems with re-colonization. Thus, decolonization strategies targeting intracellular *S. aureus* should be considered for future testing. Much is still unknown about *S. aureus* persistence in nasal tissue. Knowledge of the exact localization of *S. aureus* in nasal tissue will be important for the understanding of the host responses against *S. aureus*. Further work on the identification of bacterial and host factors can provide us with tools for targeted prevention of *S. aureus* colonisation and infection.

## Additional file

Additional file 1: Table S1. Results from *spa*-typing in the previous Tromsø 6 study and the present study. Additional documentation. (PDF 91 kb)

## Abbreviations

ATCC: American type culture collection
CFU: Colony forming unit
ClfB: Clumping factor B
CLSM: Confocal laser scanning microscopy
IsdA: Iron-regulated surface determinant
MRSA: Methicillin-resistant *Staphylococcus aureus*
MSCRAMMs: Microbial surface components recognizing adhesive matrix molecules
PBS: Phosphate buffered saline
SdrD: Serine-aspartate repeat protein D
Spa: Staphylococcal protein A
UNN: University hospital of North Norway
WTA: Wall teichoic acid
