# Supplementary material for: Localization of Staphylococcus aureus in tissue from the nasal vestibule in healthy carriers
Source: BMC Microbiol. 2017 Apr 5;17:89. doi: 10.1186/s12866-017-0997-3 (PMC5382455; doi:10.1186/s12866-017-0997-3)
Supplement: Additional file 1: Table S1. — Results from spa-typing in the previous Tromsø 6 study and the present study. Additional documentation. (PDF 91 kb) [file 12866_2017_997_MOESM1_ESM.pdf]

Table S1 Results from *spa*-typing in the previous Tromsø 6 Study and the present study.

Sample no.[a] (n=39)	Carrier status Tromsø 6 Study[b]	Two nasal swab samplings and *spa*-type Tromsø 6 Study[c]	One nasal swab sampling and *spa*-type Present study[d]
101V	Carrier	t056/t056	-
102V	Others	-	-
104H	Others	-	-
105H	Others	-	-
106	Carrier	t081/t012	-
107H	Carrier	t084/t084	t084
108H	Others	-	-
109H	Others	-	-
112H	Carrier	t012/t279	t279
113H	Others	-	-
114V	Carrier	t084/t084	t6894
115V	Others	-/t012	-
116H	Others	t065/-	-
119V	Others	-	-
120H	Others	-	-
121H	Carrier	t021/t021	t021
122V	Others	-	-
123H	Others	-	-
124H	Others	-	t084
125H	Carrier	t012/t012	t012
126H	Carrier	t122/t122	t917
127H	Carrier	t190/t190	t774
128H	Carrier	t726/t726	t726
129H	Others	-	-
131V	Others	-	-
132V	Others	-	-
133V	Others	-	-
134V	Carrier	t371/t371	t346
135V	Carrier	t002/t002	t002
137V	Carrier	t050/t050	-
138H	Others	-	-
139H	Carrier	t136/t136	t136
141V	Others	-	-
142H	Carrier	t2216/t2216	t7479
143V	Carrier	t364/t364	-
144V	Carrier	t012/t012	t246
145H	Carrier	t246/t246	t246
146H	Others	-	t362
147H	Others	-	-

[(a)]Left nostril biopsied (V); right nostril biopsied (H).
[(b)]Carrier status was defined in the Tromsø 6 Study in 2007-2008 as persistent carrier or others. Noncarriers and intermittent carriers are grouped as "others".
[(c)]Results from nasal swabbing (two samplings with median time interval of 28 days) and *spa*-typing in The Tromsø 6 Study in October 2007- August 2008; -, negative for *S. aureus*.
[(d)]Results from one nasal swab sampling and *spa*-typing in the present study in 2010-2011; -, negative for *S. aureus*.
